# Complement receptor 2 is up regulated in the spinal cord following nerve root injury and modulates the spinal cord response

**DOI:** 10.1186/s12974-015-0413-6

**Published:** 2015-10-26

**Authors:** Rickard P. F. Lindblom, Alexander Berg, Mikael Ström, Shahin Aeinehband, Cecilia A. Dominguez, Faiez Al Nimer, Nada Abdelmagid, Matthias Heinig, Johan Zelano, Karin Harnesk, Norbert Hübner, Bo Nilsson, Kristina Nilsson Ekdahl, Margarita Diez, Staffan Cullheim, Fredrik Piehl

**Affiliations:** Department of Clinical Neuroscience, Neuroimmunology Unit, Karolinska Institutet, Stockholm, Sweden; Department of Cardiothoracic Surgery and Anaesthesia, Uppsala University Hospital, Uppsala, Sweden; Department of Neuroscience, Division of Neuronal Regeneration, Karolinska Institutet, Stockholm, Sweden; Experimental Genetics of Cardiovascular Diseases, Max-Delbrück Center for Molecular Medicine, Berlin, Germany; Department of Immunology, Genetics and Pathology, Rudbeck Laboratory, Uppsala University, Uppsala, Sweden; Neuroimmunology Unit L8:04 CMM, Karolinska University Hospital, 171 76 Stockholm, Sweden

**Keywords:** Complement system, Complement receptor 2, Neuroinflammation, Neurodegeneration, Synapses

## Abstract

**Background:**

Activation of the complement system has been implicated in both acute and chronic states of neurodegeneration. However, a detailed understanding of this complex network of interacting components is still lacking.

**Methods:**

Large-scale global expression profiling in a rat F2(DAxPVG) intercross identified a strong *cis*-regulatory influence on the local expression of complement receptor 2 (Cr2) in the spinal cord after ventral root avulsion (VRA). Expression of Cr2 in the spinal cord was studied in a separate cohort of DA and PVG rats at different time-points after VRA, and also following sciatic nerve transection (SNT) in the same strains. Consequently, *Cr2*^*−/−*^ mice and Wt controls were used to further explore the role of Cr2 in the spinal cord following SNT. The in vivo experiments were complemented by astrocyte and microglia cell cultures.

**Results:**

Expression of Cr2 in naïve spinal cord was low but strongly up regulated at 5–7 days after both VRA and SNT. Levels of Cr2 expression, as well as astrocyte activation, was higher in PVG rats than DA rats following both VRA and SNT. Subsequent in vitro studies proposed astrocytes as the main source of Cr2 expression*.* A functional role for Cr2 is suggested by the finding that transgenic mice lacking *Cr2* displayed increased loss of synaptic nerve terminals following nerve injury. We also detected increased levels of soluble CR2 (sCR2) in the cerebrospinal fluid of rats following VRA.

**Conclusions:**

These results demonstrate that local expression of Cr2 in the central nervous system is part of the axotomy reaction and is suggested to modulate subsequent complement mediated effects.

**Electronic supplementary material:**

The online version of this article (doi:10.1186/s12974-015-0413-6) contains supplementary material, which is available to authorized users.

## Background

The complement system, a part of the innate arm of immunity, is activated in various inflammatory conditions [1]. A number of studies have also documented complement activity in the CNS both in acute conditions like stroke [2] and traumatic brain injury (TBI) [3], as well as in chronic diseases like multiple sclerosis (MS) [4, 5] and Alzheimer’s disease (AD) [6], all characterized by a varying degree of inflammatory features present in the tissue. The complement system consists of a large number of components, many of which are primarily produced in the liver, but some are also expressed within the central nervous system CNS [7, 8]. Activation of the complement cascade is needed for a wide range of important defense functions, including cell-lysis, chemotaxia, opsonisation, and immune cell stimulation [9, 10] but may also aggravate tissue damage [3, 5, 6, 11]. The mechanisms underlying complement activation in the CNS are complex and still not fully understood. The intricate structure of the system not only offers flexibility and efficiency, but also provides a basis for multiple points of possible dysregulation [12].A prerequisite for the agility of complement responses is a wide distribution of complement receptors, present on a range of cell types including macrophages [13], T cells [14], B cells [15], microglia, and astrocytes [16, 17]. Furthermore, many of the complement receptors exist in both membrane-bound and secreted forms, for instance, both complement receptors 1 (CR1) and 2 (CR2) exist in soluble forms: sCR1 [18] and sCR2 [19], respectively. Thus, the functional consequences of complement receptor expression depends not only on cellular localization, but also if the protein is anchored to the membrane or released into the extracellular space. Thus, soluble complement receptors can inhibit the cascade by binding complement proteins [12], a fact which has been explored in complement directed therapies [20].

We have previously described strain-dependent differences in the local expression of the upstream complement components C1q and C3 in the spinal cord of the inbred DA and PVG rat strains following a standardized nerve injury, where differences in expression of C1q correlated with subsequent nerve cell loss [21]. Furthermore, we recently identified distinct regulatory pathways of several complement components, including C1q and C3, in a F2(DAxPVG) intercross using the same nerve injury model [22]. The aim of the current study was to characterize and genetically map and characterize any possible differences in the local expression of complement receptors in this F2(DAxPVG) intercross.

## Methods

### Animals and operations

The inbred MHC congenic rat strain Dark Agouti-*RT1*^*av1*^ (DA) and the inbred MHC congenic Piebald Viral Glaxo-*RT1*^*av1*^ hereafter called PVG were bred and maintained in the in-house breeding facility under specific pathogen-free conditions and climate-controlled environment with 12 h light/dark cycles and fed standard rodent chow and water ad libitum. The F2(DAxPVG) intercross has been described previously [22–24]. In brief, DA/PVG males and females were crossed generating two groups of offspring (F1), in turn mated reciprocally generating four groups of F2 progeny from which a total of 144 animals were used. Both female and male rats and an equal number of rats from each of the four groups were studied.

All animals from the F2 intercross were at an age of 9–12 weeks subjected to unilateral avulsion of the left lumbar L3–L5 ventral roots, as described in Additional file 1. Five days post-operatively, the animals were euthanized with CO_2_ and perfused via the ascending aorta with ice-cold PBS containing heparin (LEO Pharma AB, Malmö, Sweden) (10 IE/ml). Spinal cords were dissected and examined using a dissection microscope to verify the completeness of the lesion and exclude any visible signs of hemorrhage or necrotic zones. After removal of the scar on the superficial part of the spinal cord, the ipsilateral ventral quadrant of L3, L4, and L5 was dissected out, and then snap-frozen for subsequent mRNA extraction.

The G12 (DAxPVG) advanced intercross line (AIL) was developed by continued structured breeding from a G10 AIL previously established [25]. A cohort of 161 G12 animals were subjected to ventral root avulsion (VRA), with a 5-day post-operative survival and the L3 segment used in the expression studies.

An additional cohort consisting of 72 DA and PVG rats was used to analyze the kinetics following VRA. The animals were divided into five experimental groups of 5–7 individuals with 1, 3, 5, 7, or 14 days post VRA survival and one naïve (un-operated) control group. The L3 segments were taken for mRNA preparation and the L4–L5 segments were taken en bloc and snap-frozen for further preparation/sectioning.

DA and PVG animals (*n* = 22) were operated with left sided sciatic nerve transection (SNT) below the obturator tendon. Half the group was euthanized and perfused with ice-cold PBS with heparin 5 days after operation, and the left-sided L4 segment was taken for further RT-PCR analysis, with the right L4 segment as control. The remaining group was perfused with Lana’s fixative at the same post-operative survival. These spinal cords were dissected, kept in 4 % PFA overnight, and then transferred to 10 % sucrose in PBS before mounting and sectioning of the L3–L5 segments.

*Cr2*^*−/−*^ mice (Balb/c background) were kindly provided by Professor Birgitta Heyman, Department of Medical Biochemistry and Microbiology, Uppsala University, and have previously been described [26]. Control Balb/c mice were purchased from Charles River (Wilmington, MA). *Cr2*^*−/−*^ mice and Balb/c mice (*n* = 27) were operated with left-sided SNT using the same surgical procedure as in the rats. At 5 days post-operative survival, 11 animals were euthanized with CO_2_ and perfused with PBS, with dissection of the L4 segments for RT-PCR analysis, as in the rats. At the same post-operative survival, the remaining 16 animals were perfused with Lana’s fixative and the L3–L5 segments taken as described for the SNT rats.

CSF samples were obtained from seven PVG animals, three naïve and four animals with a 5-day post-operative survival after VRA. The posterior cranial fossa was defined, and a fine needle (BD Valu-set 23G, BD Pharmingen) attached to a 1-ml syringe was used to carefully aspirate CSF from the cerebellomedullary cistern using a stereotactic frame. The CSF obtained (100–150 μl from each animal) was immediately put on dry-ice and stored at −80 °C. The CSF samples were diluted 1:4 and then analyzed for presence of rat sCD21 with sandwich ELISA technique according to the manufacturer’s instructions (USCN Life, Wuhan, China, cat.nr E0750r). All tissues were stored at −80 °C.

### DNA preparation and genotyping

Genomic DNA was extracted from rat tail tips according to methods previously described [27]. Polymorphic microsatellite markers were selected from the Rat Genome Database (http://rgd.mcw.edu) and the Ensembl database (www.ensembl.org). The F2 intercross was genotyped with 113 microsatellite markers evenly distributed across the genome, with an average distance of 20 cM based on previous knowledge of optimum spacing [28]. The successful genotyping rate was 95.3 %. Details of the F2 genotyping have been described previously [22]. The G12 intercross was densely genotyped in the region of the *Cr2* gene and the peak marker from the F2 intercross, using four microsatellite markers (D13Rat192, D13Rat159, D13Rat141, and D13Rat49), with an average marker distance of 4 cM. The *Cr2* gene is located at the end of RNO13 and all markers were located up stream of *Cr2*, which is why four markers were considered to give sufficient genomic resolution.

### RNA and cDNA preparation

RNA and cDNA was prepared using standard methodology as previously described [22].

All steps were performed under RNAse-free conditions. The tissue samples consisted of the injured L3 ventral cord quadrant from VRA animals in the kinetic study and the G12 cohort, while the corresponding L4 ventral cord quadrant was used in the F2 cohort. From the SNT animals, both mice and rats, both the ipsi- and contralateral L4 segments were used.

### RT-PCR

Real-time PCR was conducted using a three-step PCR protocol using IQ5 and CFX384 software (Bio-Rad, Hercules CA). All primers were designed with Beacon Designer 5.0 software (Bio-Rad) and tested for specificity by running the amplified product on gels with silver staining. Two house-keeping genes were used to normalize the levels of mRNA expression of the studied transcripts; hypoxanthine guanine phosphoribosyl transferase (Hprt) and glyceraldehyde 3-phosphate dehydrogenase (Gapdh). Normalized expression levels were calculated with the IQ5 or CFX 384 software. We chose C1qb as a marker for C1q expression since it was previously shown to correlate with nerve cell loss [21]. All primer sequences are presented in Tables 1 and 2. The *Cr2* gene is disrupted but not completely depleted in the *Cr2*^*−/−*^ which explains the small expression of the gene seen even in the *Cr2*^*−/−*^.

**Table 1:** Sequences for rat RT-PCR primers

Primer name	Forward sequence	Reverse sequence
Gapdh	TCAACTACATGGTCTACATGTTCCAG	TCCCATTCTCAGCCTTGACTG
Hprt	CTCATGGACTGATTATGGACAGGAC	GCAGGTCAGCAAAGAACTTATAGCC
C1qb	TCATAGAACACGAGGATTCCATACA	GACCCAGTACAGCTGCTTTGG
C3	GGGAGCCCCATGTACTCCAT	GGGACGTCACCCTGAGCAT
Gfap	AAGCACGAGGCTAATGACTATCG	AAGGACTCGTTCGTGCCG
Mrf-1	GGAGGCCTTCAAGACGAAGTAC	AGCATTCGCTTCAAGGACATAATA
CD11b	ATCCGTAAAGTAGTGAGAGAAC	TCTGCCTCAGGAATGACATC
Cr2 (CD21)	GGCTACCTTATGGCTGGAGAG	AGAGTCACAGTAGTCCCAAACC
Cr1 (CD35)	GGCTTGAGACCGCTGTGAGG	TGGATTCATCAGTTGGATTTATAGGTTTGG
CD19	CTGTTGAGGACTGGTGGATGGATAG	CTCGCTGTCTGGCTCTTCATAGG

**Table 2:** Sequences for mouse RT-PCR primers

Primer name	Forward sequence	Reverse sequence
Gapdh	TCAACTACATGGTCTACATGTTCCAG	TCCCATTCTCAGCCTTGACTG
Hprt	CTCATGGACTGATTATGGACAGGAC	GCAGGTCAGCAAAGAACTTATAGCC
C1qb	AGAGCAAGAGGAGGTTGTTCAC	GCAGGAAGATGGTGTTGGATAGG
C3	GCTGCTGTCTTCAATCACTTCATC	GCCTCTTGCCTCTTCTCTATGC
CD11b	CCCAGAGGCTCTCAGAGAATGTC	CTTCATCTTCTGAAAGTCAATGTT
Cr2 (CD21)	AACCTGGCTATTTGCTCACTGG	ACTTTCCTGGATGTTCACACTGG
Gfap	GGTAAGATGACTGAGCGGATGG	TCGTGGTAAAGACTGTGGAGATG
Mrf-1	GGAGGCCTTCAAGACGAAGTAC	AGCATTCGGTTCAAGGACATAATA

### Microarray hybridization

RNA from 144 L4 ventral cord quadrants from F2 animals were used for microarray hybridization using Affymetrix Rat gene 1.0 ST Array chips (Affymetrix, Santa Clara, CA). See Additional file 1 for details. The microarray data is available in MIAME-compliant (minimal information about a microarray experiment) format at the ArrayExpress Database (http://www.ebi.ac.uk/arrayexpress) under accession code E-MTAB-303.

### eQTL analysis and gene expression network construction

An expression quantitative trait locus (eQTL) is a gene region that regulates gene expression rather than a functional trait or disease. eQTL mapping is performed by combining expression data from global gene expression profiling with a complete linkage map, derived from whole-genome mapping using for instance microsatellite or SNP markers dispersed throughout the genome. This enables the identification of large numbers of expression QTLs and the study of co-regulated transcripts at various positions of the genome. eQTLs are classified into *cis*- or *trans*-acting according to the distance between the locations of genetic marker and the affected transcript. For a *cis*-acting QTL, the expression is regulated from the same position as the gene is located in the genome.

The current eQTL mapping has been described previously [22]. In brief, microarray gene expression data were normalized using the RMA algorithm [29], implemented in the Bioconductor package “oligo.” Raw expression intensities were background corrected, quantile normalized, log2 transformed, and summarized on the level of transcript clusters. Transcript annotation was taken from the biocondutor package “ragene10sttranscriptcluster.db.” eQTLs were mapped for all transcript clusters using the QTL reaper software [30] against the 113 genomic markers, and 10^6^ permutations were performed in order to assess genome-wide significance of eQTLs; *p* < 0.01 at genome-wide level was considered significant. The eQTLs were classified into *cis*- or *trans*-acting according to the distance between the locations of genetic marker and the affected transcript. If the distance was smaller than 20 Mb, we assumed *cis*- and otherwise *trans*-regulation. The identified Cr2 cluster (D13Rat49) was analyzed for enrichment of specific pathways and expression patterns using the Biocondutor package GOstats [31]. To enable identification of strongly connected hub genes in the eQTL gene expression network, we applied a graphical Gaussian model (GGM) and for each cluster of *trans*-regulated transcripts, we constructed gene expression networks as previously described reporting significant edges with FDR < 0.1 [32].

### Primary astrocyte and microglia cultures

Primary astrocytes and microglia were isolated from adult PVG brains of 10-week-old rats. A detailed protocol can be found in Additional file 1. The astrocytes and microglia were left unstimulated (only DMEM/F12 complete medium, supplemented with 10 % heat-inactivated FCS, penicillin-streptomycin 100 units/ml, 100 μg/ml) or stimulated with recombinant rat TNF-α (R&D Systems, Minneapolis, MN) at the concentration 20 ng/ml for 24 h after which the cells were lysed for RNA extraction and subsequent RT-PCR expressional analysis.

### Immunohistochemistry and quantification of synaptophysin immunoreactivity

Rat and mouse spinal cord sections were serially cut (14 μm) on a cryostat (Leica Microsystems, Wetzlar, Germany) at the level of the L4 segment. Detailed protocols can be found in the Additional file 1. For rat antisera directed against synaptophysin (rabbit anti-rat 1:200, Invitrogen, Carlsbad, CA), GFAP (mouse anti-rat, 1:400, Sigma), Iba1 (rabbit anti-rat, 1:200, Wako, Richmond, VA), or C3 (mouse anti-rat, 1:100 Abbiotec, San Diego, CA) was used together with appropriate flourophore-conjugated secondary antibody (Cy3 donkey anti-rabbit 1:500, Jackson Immuno Research, West Grove, PA; Alexa Fluor 488 donkey anti-rabbit, 1:150 and Alexa Fluor 594, goat anti-mouse, 1:300, both from Invitrogen). For mouse, the following antisera were used: GFAP (mouse anti-rat, 1:400, Sigma), CD68 (rat anti-mouse, 1:100, Abcam, Cambridge, UK), and CD21 (rabbit anti-mouse 1:200, Abcam) with the following secondary antibodies; Alexa 488 donkey anti-rat 1:150 and Alexa 568, donkey anti-rabbit 1:300 (Invitrogen).

Sections were examined in a Zeiss LSM 5 Pascal confocal laser scanning microscope (Carl Zeiss GmbH, Göttingen, Germany) or a Leica DM RBE microscope system (Leica). Semiquantitative measurements of immunoreactivity were carried out in ImageJ (NIH, Bethesda, MD) on confocal images. The immunoreactivity in the ventral horn of the spinal cord was compared to the corresponding contralateral side in the same spinal cord section. The images were captured in the optical plane with the maximal immunoreactivity, and all settings for compared images were identical. At least four spinal cord sections from each animal were measured, and the mean ipsilateral/contralateral signal ratio for each animal was used for statistical analysis.

### Statistical analysis

The software program R 2.6.0 was used to carry out statistical analyses and create all graphs depicting eQTL localization using the package qtl1.14-2. LOD > 3.3 corresponds to *p* < 0.0001 [33]. One-way ANOVA calculated with GraphPad Prism 5.0 (San Diego, CA) were carried out on RT-PCR and protein data; results are represented as mean ± SEM. Unpaired *t* test was used to assess differences in immunoreactivity (GraphPad Prism 5.0). Correlations between genes in the co-expression network in the F2 intercross were calculated using Pearson’s algorithm assuming equal distribution and visualized graphically using linear regression plots, also in GraphPad Prism 5.0. In general, *p* < 0.05 was considered statistically significant, except for in the microarray analysis where *p* < 0.01 was used.

### Study approval

All experiments in this study were approved by the ethical committee for animal experimentation (Stockholms Norra Djurförsöksetiska Nämnd), Stockholm, Sweden, under ethical permits N42/06, 225/08, N32/09, N343/10, and N122/11.

## Results

### eQTL mapping of complement and complement receptors in the rat spinal cord following ventral root avulsion

**Fig. 1:**
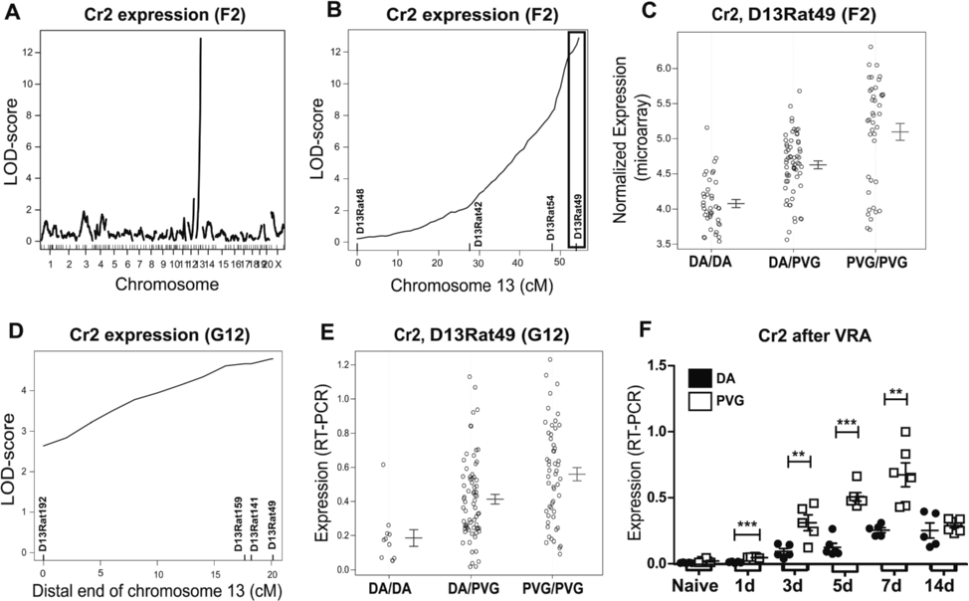
Genetic analysis of Cr2 expression following ventral root avulsion. Regulation of Cr2 expression following VRA is under strong genetic control. Global expressional profiling of injured spinal cords from a F2(DAxPVG) rat intercross (*n* = 144) reveals a *cis*-acting expression QTL at the end of chromosome 13 (**a**–**b** the *black box* marks the genetic area around D13Rat49 subsequently fine-mapped in the G12). D13Rat49 PVG alleles are associated with higher Cr2 expression (**c**). The *cis*-acting control of Cr2 expression after nerve injury was confirmed in a G12(DAxPVG) advanced intercross line (*n* = 163) (**d**). Again, PVG alleles gave rise to higher expression (**e**). A kinetic study of the injury response in DA and PVG animals with different post-operative survival following VRA revealed that Cr2 is expressed at very low levels in naïve spinal cord but strongly up regulated following injury, particular in PVG (**f**). *n* = 5–7 per strain per time-point following VRA. LOD > 3.3 corresponds to *p* < 0.0001, *p < 0.05, **p < 0.01, and ***p < 0.001. In **c** and **d**, the results are presented as mean ± SD and in **f** as mean ± SEM

By global expression profiling in an F2(DAxPVG) intercross, we recently identified new pathways regulating complement expression following a standardized nerve injury model, ventral root avulsion (VRA) [22]. This data set here was employed to analyze the expression of the complement receptors Cr1-4. In the F2 cohort (*n* = 144), differences in expression of Cr2 (also called CD21) displayed an extremely strong *cis*-regulation from an expression quantitative trait locus (eQTL) on chromosome 13. Indeed, the Cr2 transcript was one of the most strongly regulated eQTLs in the whole dataset, which contained more than 27,000 transcripts, with a logarithm of odds (LOD) score of over 13 (genome-wide *p* value <10^−6^) for the peak marker D13Rat49 located at 104.4 Mb, with higher expression driven by PVG alleles (Fig. 1a–c). *Cr2* itself is located at 111.1 Mb. Expression of the CD11b subunit of Cr3 (also called Itgam) was *cis*-regulated from D1Arb21 on chromosome 1 (187.1 Mb) (*p* value <0.001) and was higher in animals with DA alleles. Expression of the CD11c subunit of Cr4 (also called Itgax) was *cis*-regulated (*p* < 0.001) from D1Rat56, chromosome 1 at 172.9 Mb, with higher expression driven by PVG alleles. Differences in Cr1 (CD35) expression did not map anywhere in the genome. Based on these results, Cr2 was selected as a candidate for further study given the extremely strong monogenic influence regulating its expression. In mice, but not in rats or humans, *Cr1* and *Cr2* are splice variants of the same gene [34]. Since mice are used in consequent experiments, we characterized Cr1 expression in the rats for comparative purposes.

### Expression of Cr2 following VRA correlates positively with anti-inflammatory and growth promoting genes and negatively with pro-inflammatory genes

**Fig. 2:**
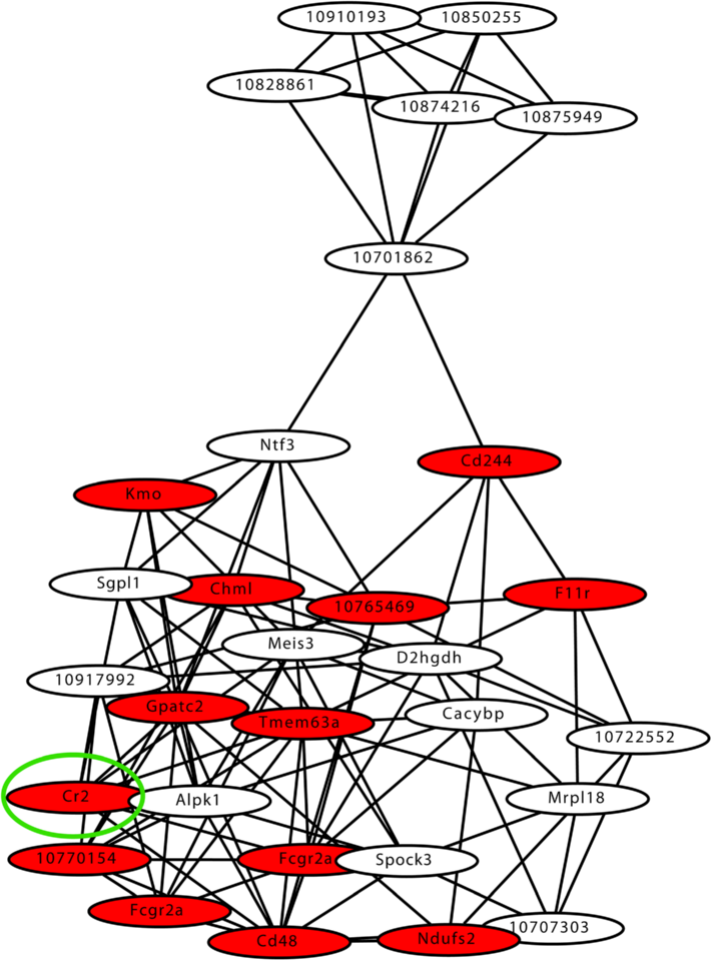
Gene expression network of transcripts regulated from D13Rat49 in the F2(DAxPVG) intercross. A co-expression gene network was constructed from all transcripts regulated from D13Rat49. All 31 genes regulated from D13Rat49 were closely interconnected; the association between the genes is based on pair-wise co-variation of expression levels. *Cis*-regulated genes are marked with *red circles*, the other genes are regulated in *trans*. The Cr2 transcript is surrounded by a *green ring*. Cr2 expression correlated negatively with expression of multiple genes with pro-inflammatory action, for example CD48, CD244, F11r, and Fcgr2a all involved in lymphocyte activation. Conversely, Cr2 expression correlated positively with Sgpl1 and Meis3, genes involved in anti-inflammatory and cellular survival processes. *n* = 144 F2(DAxPVG) rats

Thirty genes were co-regulated with Cr2 from D13Rat49, and a co-expression gene network was constructed by analyzing pair-wise co-variation in expression between all these transcripts (Fig. 2). The cluster was enriched for genes involved in leucocyte activation (*p* < 0.01) and co-factor binding (*p* < 0.001). There were multiple transcripts directly co-regulated with Cr2, where those associated with pro-inflammatory action, such as lymphocyte activation, correlated negatively with Cr2 expression, for example CD48, CD244, F11r, and Fcgr2a (Additional file 2: Table S1). Conversely, Sgpl1, recently demonstrated to have anti-inflammatory effects [35], and Meis3, involved in neuronal development [36] and cellular survival [37], displayed a positive correlation with Cr2 expression (Additional file 2: Table S1).

### Confirmation of a *cis*-acting control of Cr2 expression

Microsatellite markers in the F2 intercross were interspaced with an average distance of 20 cM. In order to both fine-map the *cis*-acting effect on Cr2 expression, as well as to reproduce the microarray expression data in an independent experiment, spinal cords from 161 animals from an advanced G12(DAxPVG) intercross subjected to VRA were analyzed with RT-PCR. High-resolution linkage analysis in the G12 animals was performed by dense microsatellite mapping around the peak marker from the F2 intercross. As the *Cr2* gene is located yet more downstream than the most distal polymorphic marker available, the observed >2 LOD-score drop corresponds to a CI of 99.8 % [38]. The replication of the finding that Cr2 expression is regulated from the region of the microsatellite marker closest to the *Cr2* gene (D13Rat49, 104.4 Mb) confirms the *cis*-regulatory effect (Fig. 1d). As in the F2 intercross, D13Rat49 PVG alleles gave rise to higher Cr2 expression (Fig. 1e).

### Kinetics of Cr1-2 expression and glial response following ventral root avulsion

The kinetics of Cr2 expression following VRA was assessed using RT-PCR in DA and PVG rats, demonstrating peak expression 7 days after injury, with significantly higher expression in PVG rats at most time-points (Fig. 1f). Cr1 expression showed a late up regulation in both strains at 14 days post-injury without major strain differences (Additional file 3: Figure S1A). Since Cr2 is highly expressed by B cells, in complex with CD19, we also determined CD19 mRNA levels. Expression of CD19 was barely measurable and without discernible strain differences, suggesting minimal infiltration of B cells (Additional file 4: Figure S2A).

Glial activation occurring after injury was assessed using glial fibrillary acidic protein (Gfap), microglia response factor-1 (Mrf-1), and CD11b (a subunit of Cr3) as markers of astrocyte and microglia activation, respectively. Expression of both Gfap (Additional file 3: Figure S1B) and Mrf-1 (Additional file 3: Figure S1C) increased in a bimodal fashion, with an initial rise at 1 and 3 days, followed by a second increase 7 days after injury. Expression of Gfap was higher in the PVG strain at 3 and 7 days after injury (Additional file 3: Figure S1B). Expression of Mrf-1 did not differ between strains, suggesting a limited strain influence on general state of microglial activation (Additional file 3: Figure S1C). In contrast, expression of CD11b was significantly increased in DA compared to PVG at most time-points, suggesting an increase of a CD11b^+^ microglia subset in the DA strain (Additional file 4: Figure S2D).

### Complement expression following sciatic nerve injury is associated with loss of synaptic terminals

**Fig. 3:**
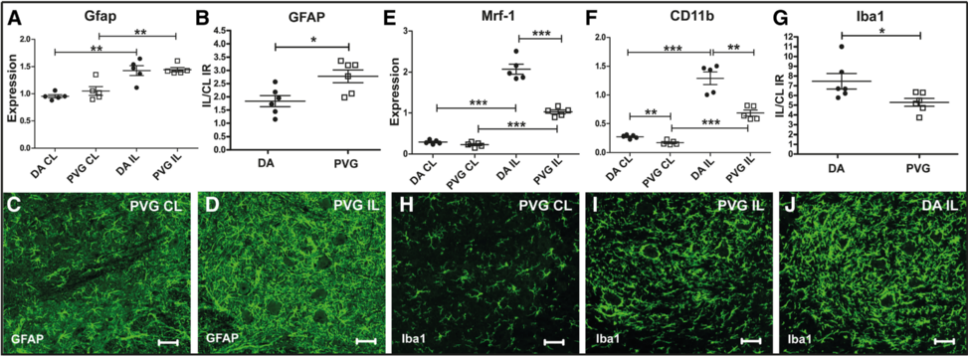
Glial activation in DA and PVG rats following sciatic nerve transection. Sciatic nerve transaction in DA and PVG rats leads to increased Gfap expression without discernible strain differences (**a**). Quantification of GFAP immunoreactivity in the dorsal spinal motor nucleus demonstrates more pronounced astrocyte activation in the PVG compared to DA strain (**b**–**d**). More intense microglia activation occurs in the DA strain following SNT, as assessed by expression of Mrf-1 and CD11b (**e**–**f**), as well as by quantifying Iba1 immunoreactivity (**g**–**j**). *IL* ipsilateral, *CL* contralateral. *Scale bar* equals 40 μm. *n* = 5 + 5 RT-PCR, 6 + 6 IHC; *p < 0.05, **p < 0.01, and ***p < 0.001. The results are represented as mean ± SEM

**Fig. 4:**
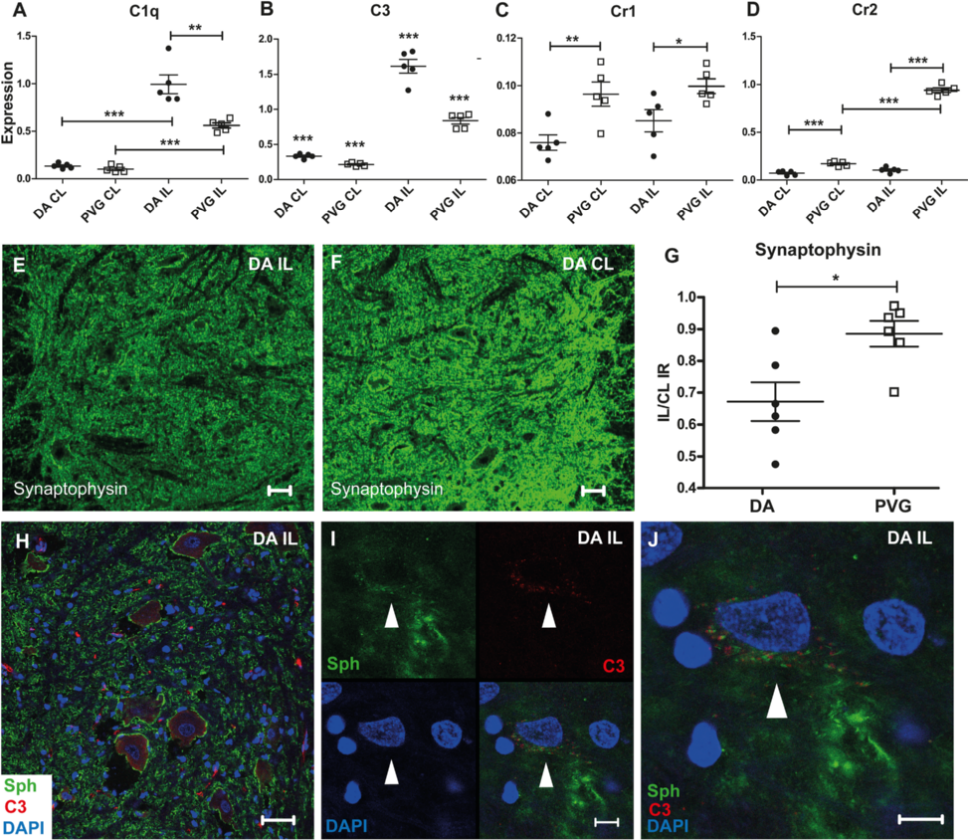
Complement expression is associated with synapse loss following sciatic nerve transection in rat. Both C1q and C3 are up regulated in DA and PVG rats following SNT but with significantly higher levels in DA (**a** and **b**). Similar to VRA, Cr1 was not regulated after injury in either strain (**c**), whereas Cr2 was strongly up regulated in PVG (**d**). Increased complement expression following SNT is associated with loss of synaptic terminals in the ventral horn, as measured by a ratio of synaptophysin immunoreactivity in the dorsal spinal motor nucleus of the injured and control sides (**e** and **f**). Loss of synapses is more pronounced in the DA strain (**g**). C3 immunoreactivity is present as a diffuse labeling as well as a more distinct signal on the cell bodies of some motor neurons (**h**). C3 labeling is found in close proximity with synaptophysin on the contour of motor neurons visualized at higher magnification (**i**–**j**). *White arrow heads* show motor neurons distinguishable by their large nuclei (**h**–**j**). *IL* ipsilateral, *CL* contralateral. *Scale bar* equals 40 μm in **e**, **f**, and **h** and 10 μm in **i** and **j**. *n* = 5 + 5 RT-PCR and 6 + 6 IHC, all at 5 days post SNT, *p < 0.05, **p < 0.01, and ***p < 0.001. The results are represented as mean ± SEM

Sciatic nerve transection (SNT) induces a local, albeit milder than after VRA, inflammatory response in the area of axotomized cell bodies within the CNS [39, 40]. DA and PVG rats were subjected to SNT with quantification of multiple transcripts in the ipsi- and contralateral side of the spinal L4 level 5 days after surgery. Expression of Gfap was more pronounced in the PVG strain (Fig. 3a–d), while microglial markers Mrf-1 and CD11b were higher in DA (Fig. 3e, f), also confirmed by more intense Iba1 immunolabeling (Fig. 3g–j). Expression of CD19 was not increased after SNT, but levels were generally higher in DA (Additional file 4: Figure S2B). Complement expression is associated with loss of synaptic nerve terminals following both VRA and SNT [22, 41]. C1q and C3 were both up regulated following SNT, with higher expression in DA compared to PVG rats (Fig. 4a, b). Expression of Cr1 was not affected in either strain after injury, but levels were generally higher in PVG (Fig. 4c). In contrast, Cr2 displayed a conspicuous up regulation in PVG but not in DA (Fig. 4d). Quantification of synaptic density performed 5 days post SNT demonstrated a more pronounced loss of synaptic nerve terminals in DA compared to PVG rats (Fig. 4e–g). Double labeling with antisera against C3 and synaptophysin showed C3 deposition in close proximity to synapthopysin in juxtaposition to axotomized motor neurons (Fig. 4h–j).

### Cr2 protects from synaptic loss following sciatic nerve transection

**Fig. 5:**
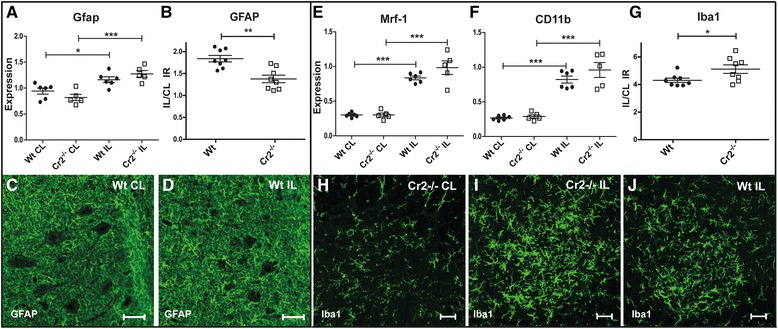
Glial activation in Balb/c and Balb/c *Cr2* ^*−/−*^ mice following sciatic nerve transection. Expression of Gfap increased in the spinal cords of both Balb/c wild-type (WT) and *Cr2* ^*−/−*^ mice following SNT but without strain differences (**a**). Quantification of GFAP immunoreactivity the dorsal spinal motor nucleus demonstrates more pronounced astrocyte activation in WT compared to *Cr2* ^*−/−*^ (**b**–**d**). Expression of Mrf-1 and CD11b are strongly up regulated in both WT and *Cr2* ^*−/−*^, without significant differences between strains at the mRNA level (**e**–**f**). In contrast, quantification of Iba1 in the dorsal spinal motor nucleus demonstrates increased immunolabeling signal in *Cr2* ^*−/−*^ compared to WT thus opposite to GFAP (**g**–**j**). *IL* ipsilateral, *CL* contralateral. *Scale bar* equals 40 μm. *n* = 6 + 5 RT-PCR, 8 + 8 IHC, *p < 0.05, **p < 0.01, and ***p < 0.001. The results are represented as mean ± SEM

As CR2 is known to bind different C3 fragments [42] and C3 was up regulated after injury, we sought to identify a functional role of the increased Cr2 expression after nerve injury. Thus, Balb/c and *Cr2*^*−/−*^ mice on Balb/c background were subjected to SNT. There was no difference in the up regulation of Gfap expression between wild type and *Cr2*^*−/−*^ animals 5 days after surgery (Fig. 5a), but GFAP immunoreactivity was increased in wild type compared to *Cr2*^*−/−*^ (Fig. 5b–d). Both strains displayed up regulation of Mrf-1 and CD11b, as signs of microglia activation (Fig. 5e, f), with a trend for higher expression levels of both genes and significantly more intense Iba1 immunolabeling in *Cr2*^*−/−*^ mice (Fig. 5g–j).

**Fig. 6:**
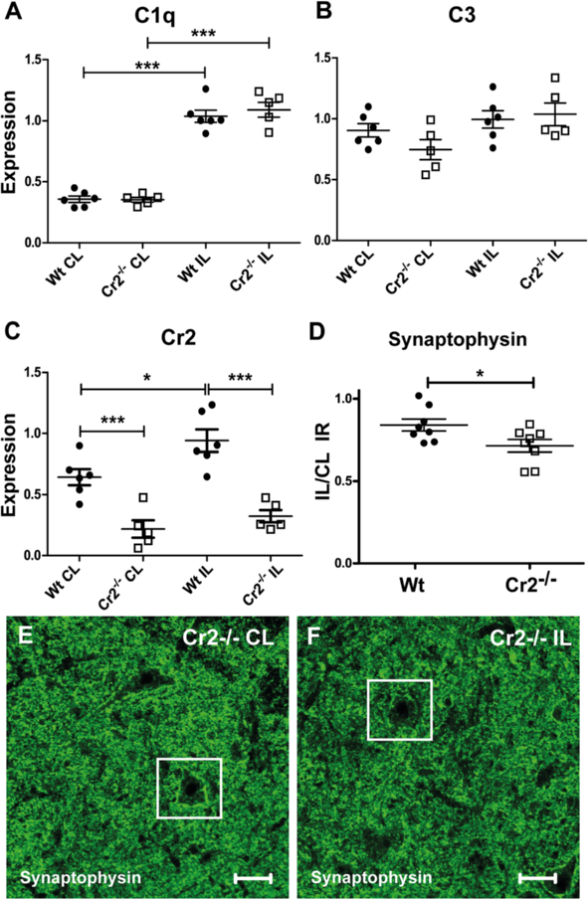
Complement receptor 2 protects against synapse loss following sciatic nerve transection. Expression of C1q is up regulated in mice following SNT, without discernible strain differences (**a**). C3 demonstrates a strong trend towards increased expression in the injured side ofthe cord in *Cr2* ^*−/−*^ but not WT mice (*p* = 0.048, *t* test), but does not quite reach significance (**b**). Cr2 is significantly up regulated following injury in WT mice (**c**). Assessment of synaptic density by quantification of synaptophysin immunolabeling signal demonstrates increased loss in *Cr2* ^*−/−*^, suggesting that CR2 protects from synapse elimination following nerve injury (**d**–**f**). The *white box* shows a motor neuron on the control and injured sides, respectively, where the dense synaptic synaptophysin-labeled aura surrounding the cell body is lost on the axotomized cell (**e** and **f**). *IL* ipsilateral, *CL* contralateral. *Scale bar* equals 40 μm. *n* = 6 + 5 RT-PCR, 8 + 8 IHC; *p < 0.05, **p < 0.01, and ***p < 0.001. The results are represented as mean ± SEM

C1q was strongly up regulated in both strains (Fig. 6a), while C3 tended to be up regulated solely in the *Cr2*^*−/−*^ strain (*p* = 0.048, *t* test) (Fig. 6b). As expected, expression of Cr2 was significantly up regulated in wild-type Balb/c mice following SNT but not in *Cr2*^*−/−*^ (Fig. 6c). Assessment of synaptic density in the dorsolateral part of the ventral horn revealed significantly increased loss of synaptophysin immunoreactivity following injury in *Cr2*^*−/−*^ compared to wild type, suggesting a more pronounced loss of synaptic nerve terminals in the absence of CR2 (Fig. 6d–f).

### Cr2 is expressed by astrocytes

**Fig. 7:**
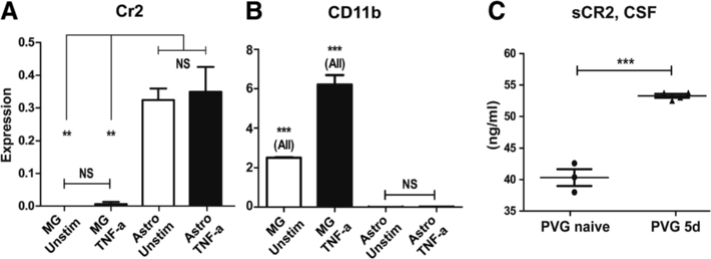
Cr2 is expressed by astrocytes in vitro, and levels of soluble CR2 are increased in cerebrospinal fluid of rats following ventral root avulsion. The expression pattern of Cr2 was similar to that of Gfap suggesting astrocytes as a likely source. To confirm that astrocytes and not microglia were the main source of Cr2 expression, cultures of both glia types were generated from adult PVG rats and stimulated with TNF-α. Cr2 expression was not detectable in microglia, whereas Cr2 was expressed by astrocytes although TNF-α stimulation did not lead to a significant increase in levels (**a**). CD11b expression was 100- to 1000-fold higher in microglia as compared to astrocytes and increased following TNF-α stimulation (**b**). CSF was collected from PVG rats subjected to VRA. Levels of soluble CR2, sCR2, were increased in injured compared to healthy rats, as measured with Elisa (**c**). *p < 0.05, **p < 0.01, and ***p < 0.001, the in vitro experiments were repeated three times with equal results. The results are represented as mean ± SEM

In a recent study, 13 antibodies specific for CR2 were evaluated for immunohistochemistry in the CNS; however, none yielded distinct stainings [43]. This is in line with our experience, as also our immunohistochemical assessments of CR2 gave a diffuse staining pattern (not shown). Previous data has suggested astrocytes as the source of CR2 in the CNS [17, 43]. We therefore established cultures of rat astrocytes, as well as microglia which are known to express CR3/CD11b [44], to assess comparative expression patterns. Cultured cells were left unstimulated or stimulated with TNF-α with determination of Cr2 expression assessed with RT-PCR. Cr2 expression was readily detectable in astrocytes, but not microglia, and was not affected by TNF-α (Fig. 7a). In contrast, CD11b expression was much higher in microglia compared to astrocytes, and increased following TNF-α stimulation (Fig. 7b).

### Soluble CR2 can be detected in CSF of rats following ventral root avulsion

CR2 also exists in a soluble form, sCR2 (sCD21), present in blood [45–48]. The existence of Cr2 in the CNS, as demonstrated by RT-PCR, in combination with the difficulty of staining for CR2 prompted us to investigate the presence of sCR2 also in the CSF. Protein ELISA measurements of sCR2 in CSF from naive and VRA-operated PVG animals showed detectable levels, with up regulation following injury, in concordance with expression data (Fig. 7c).

## Discussion

Using an unbiased genetic approach, we find here a strong naturally occurring *cis*-acting genetic influence on the local expression of Cr2 in the spinal cord following nerve root injury. Our experimental data support the notion that CR2, previously mostly studied in context of B cell biology, is an integral part of the inherent CNS response to injury and that it may modulate synaptic plasticity by regulating upstream complement activity. Hypothetically, this could be done by secreted/shed soluble CR2.

Mapping of the genetic effect was carried out in a standardized nerve injury model, VRA, where motor axons are severed at the interface between the peripheral and central nervous systems, resulting in substantial loss of injured nerve cells. For technical reasons, a more peripheral nerve injury model, SNT, was used in mice. SNT also induces inflammatory activation of microglia and astrocytes in the vicinity of the lesioned motor neurons, albeit with more limited loss of axotomized cells than VRA [40, 49, 50]. The so-called axon reaction has been a subject of study for more than a century, initially focusing on the characteristic morphological changes occurring in injured cells [51]. Subsequent research has shown alterations in electrical activity at the level of the cell body believed to be the caused by elimination of afferent synaptic input to lesioned neurons [52]. After acute mechanical injuries, the elimination of synaptic input has been assumed to be beneficial, perhaps by limiting excitotoxic stress [50, 53]. However, more recent studies suggest that reduced loss of synapses after SNT or VRA is associated with improved functional outcome [41, 54]. Likely, the molecular systems regulating plasticity of nerve terminals have to be finely tuned as excessive loss of synaptic connectivity may be an important biological substrate for chronic neurodegenerative diseases, which has been demonstrated to occur as an early disease-related phenomenon [55, 56]. An increasing body of evidence suggests that the complement system plays an important role for synaptic plasticity. Thus, upstream complement proteins such as C1q and C3 mediate synaptic remodeling during development [57, 58]. They are also implicated in normal aging, since a dramatic increase in C1q occurs in the brain of old mice and humans [59], as well as in neurodegenerative diseases such as AD and Parkinson’s disease, and also MS [6, 60]. The notion that dysregulation of the complement system occurs upstream rather than downstream of an inherent neurodegenerative process is supported by the observation that genetic variability in both *Clusterin* and *CR1* is associated to risk of late-onset AD [61, 62]. Although the functional consequences of complement activation in chronic neurodegenerative disease need to be defined more in detail, it seems plausible that excessive activation may exaggerate a neurodegenerative process.

The recent genetic association of *CR1* to AD [62] led us to examine any possible naturally occurring strain differences in expression of the four most studied complement receptors, Cr1-4, after nerve injury. Cr2-4 could all be shown to be regulated from distinct genetic regions, with the most striking finding for Cr2, which was subject to a very strong monogenic *cis*-acting regulation. In contrast, Cr1 was not regulated by injury in the rat.

CR2 has been studied mostly in context of B cell immunology, where it is expressed on mature B cells and forms a complex with CD19 and CD81. This tri-molecular formation functions as a co-receptor complex, where CR2 binds opsonized C3d and antigen-bound IgM resulting in an enhanced antigenic B cell response [63]. However, it is unlikely that B cells cause the observed strain differences in Cr2 expression due to the low expression of its B cell binding partners. The role of CR2 in the CNS has received little attention, but expression of CR2 has been previously reported on activated astrocytes and has been demonstrated to regulate neurogenesis in the mouse [17, 43].

Microglia are suggested to be involved in the emoval of synapses occurring after CNS injury, and complement is known to increase their phagocytic properties [58, 64]. In the rat strains studied here, the microglia response was stronger in DA than PVG after both SNT and VRA. Interestingly, only CD11b (Cr3), but not Mrf-1, displayed higher levels in DA, suggesting the possible existence of microglia subsets with different phagocytic abilities. This may have functional implications as mice deficient for *Cr3* (CD11b/CD18) display reduced loss of synapses during development of the visual system [58].

The similar expression pattern of Cr2 and Gfap, and the Cr2 expression in the astrocyte cultures suggest astrocytes as a source of locally expressed Cr2 in the CNS. The existence primarily of a soluble form could explain the difficulty to stain for CR2 in the CNS [43]. We examined this by measuring sCR2 levels with Elisa in CSF, which verified the presence of soluble CR2 and that levels increased following injury. The increase in sCR2 may not only reflect increased transcription, but can be the result of increased shedding of the CR2 ectodomain, which corresponds to sCR2, due to oxidative stress triggered by VRA [24, 65].

The results presented herein suggest that activated PVG astrocytes produce more CR2/sCR2, in turn of possible relevance for protecting the integrity of synaptic networks. This interpretation is supported by results obtained in *Cr2*^*−/−*^ mice, which display increased loss of synaptophysin-labeled synaptic terminals after SNT. Possibly, deficiency in CR2 also affects glial activation after nerve injury, with enhanced microglia and reduced astrocyte activation, as supported by quantification of the microglia immunolabeling signal in the axotomized sciatic motor pool. This is also supported by the constructed gene expression network, where Cr2 correlates positively with anti-inflammatory and negatively with pro-inflammatory transcripts, and by previous findings, where sCR2 was shown to modulate monocyte activity [66]. CR2 binds to C3dg and iC3b, both breakdown products of C3 [67]. Thus, increased sCR2 after injury may serve a regulatory role as a binder of deposited C3 fragments in the area of inflammation. However, it is also possible that CR2 could have a more complex role in altering the balance or activation steps of the C3 activation/cleavage cascade, with multiple active breakdown products. For instance, iC3b is the primary ligand for CR3 (CD11b/CD18) [68], whereas C3b is not bound by CR3 [69], but instead by CR1 [70]. This suggests that increased levels of sCR2 in context of nerve injury leads to decreased iC3b generation, which in turn attenuates the process of synaptic loss, as the CR3 (CD11b/CD18) positive microglia have been shown to be involved in removal of synapses [58]. It could also explain the increased microglia activation seen in the Cr2^−/−^ mice, as iC3b binding to CR3 can contribute to increased microglia activation [44].

The complexity of complement activity is illustrated by a recent study, which may at first seem contradictory to ours, since *Cr2*^*−/−*^ mice demonstrated improved initial outcome after experimental TBI [71]. However, this model differs considerably from the ones used here, since in TBI there is a dramatic loss of blood-brain barrier function leading to influx of immune cells and complement proteins from the systemic circulation. Furthermore, it is difficult to dissociate the effect of targeting both CR1 and CR2, since in mice, but not in humans and rats, the two proteins are coded in the same gene. It should also be underscored that the presence or expression of complement proteins is not synonymous with complement activation, as defined by the release of complement protein fragments with immune signaling properties and activation of the complement cascade [12]. Lastly, it is also of importance to note the different roles of soluble as compared to membrane bound forms of complement receptors, where they in one form may be activating and in the other regulatory.

## Conclusions

We identify here a strong naturally occurring genetic variation acting on *Cr2* in the rat, which affects the local expression of Cr2 in the spinal cord following both proximal and peripheral nerve injury as well as the levels of sCR2 in CSF. In mice lacking *Cr2*, peripheral nerve injury leads to increased microglia activation and more pronounced loss of nerve terminals. These results suggest a role for CR2 in regulating complement-mediated effects in the CNS. The role of CR2/sCR2 may also be relevant to explore in other settings of injury to the CNS as complement expression and/or activation is a common feature in several both acute and chronic CNS diseases.

## Additional files

Additional file 1. Additional information. (DOC 42 kb) Additional file 2: Table S1. Genes in the co-expression network regulated from D13Rat49. (DOC 61 kb) Additional file 3: Figure S1. Glial activation in DA and PVG rats following ventral root avulsion. In mice, *Cr1* and *Cr2* are splice variants of the same gene, and for completeness, Cr1 expression was studied following VRA, which revealed no consistent strain differences even if expression was higher in PVG rats at a single time point, 7 days after injury (A). Both astrocyte and microglia activation after VRA is biphasic, with most pronounced activation of both glia types at 7 and 14 days after injury, demonstrated with both RT-PCR (B–D) and immunohistochemistry (E–J). Gfap expression is higher in PVG than DA rats at both 3 and 7 days after VRA (B). Mrf-1 expression (C) and Iba1 immunoreactivity (H–J) were similar between the strains, arguing against major differences in general microglia activation. However, expression of CD11b, which could represent a subset of microglia, was higher in DA than PVG at most time-points following injury (D). *IL* ipsilateral, *CL* contralateral. Scale bar equals 40 μm. *n* = 5–7 per strain per time-point. **p* < 0.05, ***p* < 0.01, and ****p* < 0.001. The results are represented as mean ± SEM. (TIFF 16335 kb) Additional file 4: Figure S2. Low CD19 expression following ventral root avulsion and sciatic nerve transection in rats argues against B cell infiltration. A slight up regulation of CD19 occurred after VRA injury in both DA and PVG rats, but without significant strain differences, though levels tended to be higher in DA (A) and thus opposite to the expression pattern of Cr2, *n* = 5–7 per strain per time-point. No difference in CD19 expression was evident between injured and control sides after SNT (B), *n* = 5 + 5. The results are represented as mean ± SEM. (TIFF 207 kb)

## Abbreviations

CR2: complement receptor 2
CSF: cerebrospinal fluid
SNT: sciatic nerve transection
VRA: ventral root avulsion
