# Supplementary material for: Complement receptor 2 is up regulated in the spinal cord following nerve root injury and modulates the spinal cord response
Source: J Neuroinflammation. 2015 Oct 26;12:192. doi: 10.1186/s12974-015-0413-6 (PMC4624364; doi:10.1186/s12974-015-0413-6)
Supplement: Additional file 1. — Additional information. (DOC 42 kb) [file 12974_2015_413_MOESM1_ESM.doc]

# Additional file 1

# Additional information

## Material and Methods

### Ventral Root Avulsion

Unilateral avulsion of the left lumbar L3-L5 ventral roots under standardized conditions in deep isoflurane anesthesia. A dorsal laminectomy was performed at the level of the L2-L3 vertebrae. The dura was delicately opened with the point of a needle and by gently moving the sensory nerve roots the L3-L5 ventral roots were identified and avulsed from the spinal cord using a micro-forceps. The muscles and skin were then sutured in layers. Post-operative analgesia (Buprenorfin, 0.1ml, 0.3 mg/ml, RB Pharmaceuticals, Slough, England) was given subcutaneously twice daily for three days.

### Microarray hybridization

RNA from 144 L4 ventral cord quadrants from F2 animals were used for microarray hybridization performed at the Bioinformatics and Expression Analysis Core Facility (BEA) facility of Karolinska Institutet using Affymetrix Rat gene 1.0 ST Array chips (Affymetrix, Santa Clara, CA). 100 ng of total RNA was used in the first-round synthesis of double-stranded cDNA. RNA was reverse transcribed using a whole transcript cDNA synthesis and amplification kit (Affymetrix UK Ltd., High Wycombe, UK). The resulting biotin-labeled cRNA was purified using an IVT clean-up kit (Affymetrix) and quantified using a NanoDrop ND-1000 Spectrophotometer (A260/280; NanoDrop Technologies, Wilmington, DE). An aliquot (5.5 μg) of cRNA was fragmented by heat and ion-mediated hydrolysis at 94°C for 35 minutes. Confirmation of RNA quality was assessed using the Agilent 2100 Bioanalyzer (Agilent Technologies, Santa Clara, CA). Target labelling array hybridization, washing and staining was performed as described in the GeneChip Whole Transcript (WT) Sense Target Labeling manual (http://www.affymetrix.com). Arrays were scanned using the GeneChip Scanner 3000 7G and the GeneChip Command Console (Affymetrix) with default setup. 2 µg of total RNA was used for each sample. Probe details can be found at the Affymetrix website ([http://www.affymetrix.com](http://www.affymetrix.com/)). The microarray data is available in MIAME-compliant (minimal information about a microarray experiments) format at the ArrayExpress Database (<http://www.ebi.ac.uk/arrayexpress>) under accession code E-MTAB-303.

### Primary Astrocyte and Microglia cultures

Primary astrocytes and microglia were isolated from adult PVG brains of 10 week old rats perfused via the ascending aorta with ice-cold PBS containing Heparin (LEO Pharma AB, Malmö, Sweden) (10 IE/ml). After removing the meninges and the cerebellum the brains were homogenized in enzymatic solution (116 mM NaCl, 5.4 mM KCl, 26 mM NaHCO3, 1 mM NaH_2_PO4, 1.5 mM CaCl_2_, 1 mM MgSO_4_, 0.5 mM EDTA, 25 mM glucose, 1 mM cysteine and 20 u/ml papain (all from Sigma) using a micro-scissor. The homogenate was incubated for 60 min with gentle stirring at 37°C, 5% CO_2_. Next, the digested tissue was transferred to a 50 ml conical tube with stopping of the enzymatic reaction by adding HBSS (Invitrogen, Sweden) with 10% foetal calf serum, (FCS). The homogenate was spun down at 200 g for 7 min and the pellet resuspended in 2 ml of 0.5 mg/ml DNaseI (Roche, Sweden) in HBSS, then filtered through a 40 μm strainer (Becton Dickinson, Sweden) and transferred to 20 ml of 20% stock isotonic Percoll in HBSS. Another 20 ml pure HBSS was carefully added on top to create a Percoll gradient, the samples were then gently centrifuged at 200g for 20 min. The resulting pellet was washed once in HBSS and the cells resuspended in DMEM/F12 complete medium, supplemented with 10% heat-inactivated FCS, Penicillin-Streptomycin (100 u/ml, 100 μg/ml) 2 mM L-glutamine (Life Technologies) and 30% M-CSF conditioned L929 cell line supernatant and plated in 75cc tissue flasks (Sarstedt, Nümbrecht, Germany) coated with poly-l-lysine (Sigma) and incubated at 37°C and 5% CO_2_ in a humidified incubator. Medium was changed twice weekly until the cells became confluent (approximately 14 days). When full confluence of the cell layer was reached, the mixed glial cells were harvested using pre-warmed trypsin (Gibco).

To separate microglia and astrocytes from each other in the mixed glial cell culture, magnetic separation was used according to the manufacturer's instructions. In brief, the mixed cell suspension was centrifuged at 300g for 10 min. The cell pellet was resuspended in MACS buffer (Miltenyi Biotec GmbH, Germany) and stained with PE-conjugated mouse anti-rat CD11b (BD Biosciences, Sweden) for 10 min. Then, the cells were washed and resuspended in MACS buffer followed by with AutoMACS magnetic separation using anti-PE MicroBeads (Miltenyi). The resulting microglia (CD11^+^ cells) and remaining cells (astrocytes) were seeded in 24-well plates (2 × 10^5^ cells/well and 3 × 10^5^ cells/well, respectively). The purities of microglia and astrocytes were checked with flow cytometry as described above using PE-labeled mouse anti-rat CD11b and FITC labeled mouse anti-rat GFAP antibodies (both from BD Pharmingen, Franklin Lakes, NJ). The purities of microglia and astrocytes were consistently above 95% and the reproducibility of the sorting has been demonstrated previously .

The cells (microglia and astrocytes) were then left unstimulated (only DMEM/F12 complete medium, supplemented with 10% heat-inactivated FCS, Penicillin-Streptomycin 100 u/ml, 100 μg/ml), or stimulated with recombinant rat TNF-α (R&D Systems, Minneapolis, MN) at 20 ng/ml for 24 h after which the cells were lysed for RNA extraction and subsequent RT-PCR expressional analysis.

### Immunohistochemistry and Quantification of synaptophysin immunoreactivity

Rat and mouse spinal cord sections were serially cut (14 μm) on a cryostat (Leica Microsystems, Wetzlar, Germany) at the level of L4 segment. Sections were thawed onto Superfrost plus microscope slides (Menzel-Gläser, Braunschweig, Germany) and stored at -20°C until further processing for immunohistochemistry. Sections were either post-fixed in 4% formaldehyde and 0.4% picric acid in 0.16 M phosphate buffer (pH 7.2) for 2 hours at room temperature (RT), rinsed in PBS and incubated overnight at 4˚C with primary antisera directed against Synaptophysin (rabbit anti-rat 1:200, Invitrogen, Carlsbad, CA), or post-fixed for 30 min in 4% formaldehyde at RT before incubation with the following antibodies; GFAP (mouse anti-rat, 1:400, Sigma), Iba1 (rabbit anti-rat, 1:200, Wako, Richmond, VA), C3 (mouse anti-rat, 1:100 Abbiotec, San Diego, CA), then rinsed in PBS, incubated for 60 min with appropriate flourophore-conjugated secondary antibody (Cy3 donkey anti-rabbit 1:500, Jackson Immuno Research, West Grove, PA; Alexa Fluor 488 donkey-anti rabbit, 1:150 and Alexa Fluor 594, goat anti-mouse, 1:300, both from Invitrogen), diluted in PBS and 0.3% Triton X-100, then rinsed in PBS and mounted in PBS-glycerol (1:3). The mouse sections were processed in the same way, but the following antisera; GFAP (mouse anti-rat, 1:400, Sigma-Aldrich, St Louis, MO), CD68 (rat anti-mouse, 1:100, Abcam, Cambridge, UK) and CD21 (rabbit-anti mouse 1:200, Abcam) and the following secondary antibodies; Alexa 488 donkey anti-rat 1:150 and Alexa 568, donkey anti-rabbit 1:300 (Invitrogen).

Sections were examined in a Zeiss LSM 5 Pascal confocal laser scanning microscope (Carl Zeiss GmbH, Göttingen, Germany) or a Leica DM RBE microscope system (Leica). Semiquantitative measurements of immunoreactivity were carried out in ImageJ (NIH, Bethesda, MD) on confocal images. The immunoreactivity in the ventral horn of the spinal cord was compared to the corresponding contralateral side in the same spinal cord section. The images were captured in the optical plane with the maximal immunoreactivity and all settings for compared images were identical. At least four spinal cord sections from each animal were measured and the mean ipsilateral/contralateral signal ratio for each animal was used for statistical analysis.

## References

1. Lindblom RP, Strom M, Heinig M, Al Nimer F, Aeinehband S, Berg A, Dominguez CA, Vijayaraghavan S, Zhang XM, Harnesk K, et al: **Unbiased Expression Mapping Identifies a Link between the Complement and Cholinergic Systems in the Rat Central Nervous System.** *Journal of immunology* 2013.

2. Lindblom RP, Aeinehband S, Parsa R, Strom M, Al Nimer F, Zhang XM, Dominguez CA, Flytzani S, Diez M, Piehl F: **Genetic variability in the rat Aplec C-type lectin gene cluster regulates lymphocyte trafficking and motor neuron survival after traumatic nerve root injury.** *Journal of neuroinflammation* 2013, **10:**60.
