# Supplementary material for: Complement receptor 2 is up regulated in the spinal cord following nerve root injury and modulates the spinal cord response
Source: J Neuroinflammation. 2015 Oct 26;12:192. doi: 10.1186/s12974-015-0413-6 (PMC4624364; doi:10.1186/s12974-015-0413-6)
Supplement: Additional file 2: Table S1. — Genes in the co-expression network regulated from D13Rat49. (DOC 61 kb) [file 12974_2015_413_MOESM2_ESM.doc]

**Additional table 1. Genes in the co-expression network regulated from D13Rat49.**

| **TranscriptID** | **Gene name** | **R^2^** | **P-value** | **Dev. from zero** | **Slope** | **cis/trans** |
| --- | --- | --- | --- | --- | --- | --- |
| 10701862 | ENSRNOG00000030501 | 0,03408 | 0,0268 | **Significant** | -7,807 | trans |
| 10707303 | similar to integral membrane protein GDD1 | 0,03499 | 0,0248 | **Significant** | 8,391 | trans |
| 10722552 | myotubularin related protein 15 | 0,06061 | 0,0029 | **Significant** | 7,396 | trans |
| 10765469 | SH2 domain containing 1B | 0,0587 | 0,0034 | **Significant** | -4,599 | cis |
| 10874216 | ENSRNOG00000030501 | 0,02121 | 0,0816 | Not Significant | -6,011 | trans |
| 10875949 | ENSRNOG00000031319 | 0,02121 | 0,0816 | Not Significant | -6,011 | trans |
| 10910193 | ENSRNOG00000033798 | 0,02121 | 0,0816 | Not Significant | -6,011 | trans |
| 10917992 | ENSRNOG00000029359 | 0,2213 | <0,0001 | **Significant** | -4,159 | trans |
| Alpk1 | alpha-kinase 1 | 0,01592 | 0,1318 | Not Significant | 20 | trans |
| Cacybp | calcyclin binding protein | 0,08531 | 0,0004 | **Significant** | -8,19 | trans |
| CD244 | Cd244 molecule, natural killer cell receptor 2B4 | 0,03709 | 0,0207 | **Significant** | -7,468 | cis |
| CD48 | Cd48 molecule | 0,02821 | 0,0442 | **Significant** | -13,39 | cis |
| Chml | choroideremia-like (Rab escort protein 2) | 0,0236 | 0,066 | Not Significant | -12,39 | cis |
| D2hgdh | D-2-hydroxyglutarate dehydrogenase | 0,06427 | 0,0022 | **Significant** | 8,84 | trans |
| F11r | F11 receptor | 0,03991 | 0,0164 | **Significant** | -7,261 | cis |
| Fcgr2a (1) | Fc fragment of IgG, low affinity IIb, receptor (CD32) | 0,04473 | 0,0109 | **Significant** | -11,73 | cis |
| Fcgr2a (2) | Fc fragment of IgG, low affinity IIb, receptor (CD32) | 0,154 | <0,0001 | **Significant** | -3,747 | cis |
| Gpatc2 | G patch domain containing 2 | 0,07842 | 0,0007 | **Significant** | 7,828 | cis |
| Kmo | kynurenine 3-monooxygenase | 0,04464 | 0,011 | **Significant** | -9,693 | cis |
| Meis3 | Meis homeobox 3 | 0,08157 | 0,0005 | **Significant** | 8,646 | trans |
| Mrpl18 | mitochondrial ribosomal protein L18 | 0,0205 | 0,0869 | Not Significant | -9,009 | trans |
| Ndufs2 | NADH dehydrogenase (ubiquinone) Fe-S protein 2 | 0,02121 | 0,0815 | Not Significant | -18,86 | cis |
| Ntf3 | neurotrophin 3 | 0,01772 | 0,1118 | Not Significant | 15,7 | trans |
| Opn3 | Opsin 3 | 0,06198 | 0,0026 | **Significant** | -6,996 | cis |
| Sgpl1 | sphingosine-1-phosphate lyase 1 | 0,1173 | <0,0001 | **Significant** | 9,629 | trans |
| Spock3 | sparc/osteonectin, cwcv and kazal-like domains proteoglycan 3 | 0,001759 | 0,6177 | Not Significant | -64,89 | trans |

**Additional table 1.** Correlation with Cr2 expression of all genes in the network regulated from D13Rat49. Several proinflammatory genes, for example CD48, CD244, F11r and Fcgr2a correlate negatively with CR2 expression, whereas, Sgpl1, with anti-inflammatory effects and Meis3, involved in cellular survival displays a positive correlation with Cr2. Green rows correlate positively and red negatively with Cr2 expression.
