# Supplementary figures and images for: Complement receptor 2 is up regulated in the spinal cord following nerve root injury and modulates the spinal cord response
Source: J Neuroinflammation. 2015 Oct 26;12:192. doi: 10.1186/s12974-015-0413-6 (PMC4624364; doi:10.1186/s12974-015-0413-6)

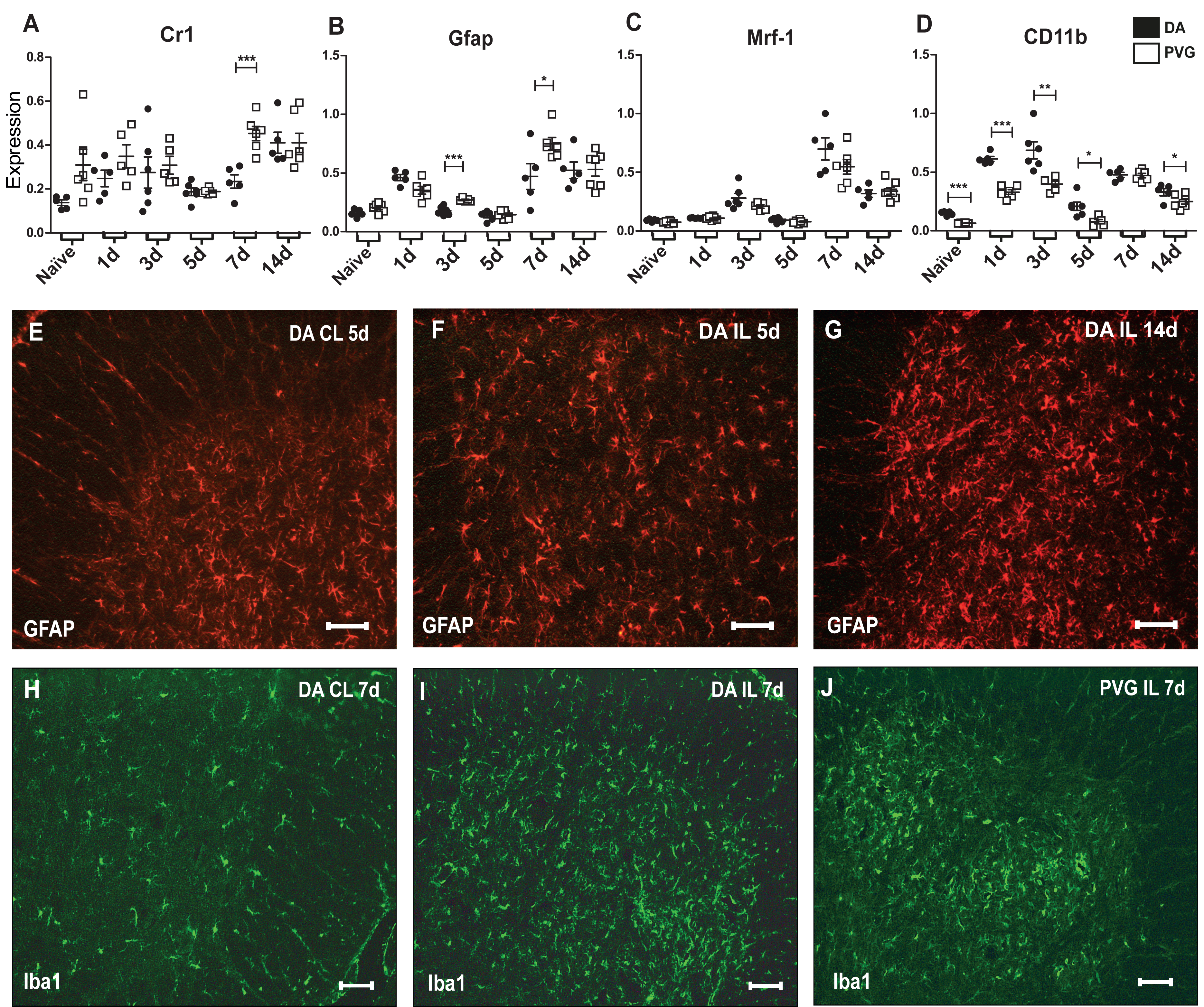

Supplement: Additional file 3: Figure S1. — Glial activation in DA and PVG rats following ventral root avulsion. In mice, Cr1 and Cr2 are splice variants of the same gene, and for completeness, Cr1 expression was studied following VRA, which revealed no consistent strain differences even if expression was higher in PVG rats at a single time point, 7 days after injury (A). Both astrocyte and microglia activation after VRA is biphasic, with most pronounced activation of both glia types at 7 and 14 days after injury, demonstrated with both RT-PCR (B–D) and immunohistochemistry (E–J). Gfap expression is higher in PVG than DA rats at both 3 and 7 days after VRA (B). Mrf-1 expression (C) and Iba1 immunoreactivity (H–J) were similar between the strains, arguing against major differences in general microglia activation. However, expression of CD11b, which could represent a subset of microglia, was higher in DA than PVG at most time-points following injury (D). IL ipsilateral, CL contralateral. Scale bar equals 40 μm. n = 5–7 per strain per time-point. *p < 0.05, **p < 0.01, and ***p < 0.001. The results are represented as mean ± SEM. (TIFF 16335 kb) [file 12974_2015_413_MOESM3_ESM.tif]

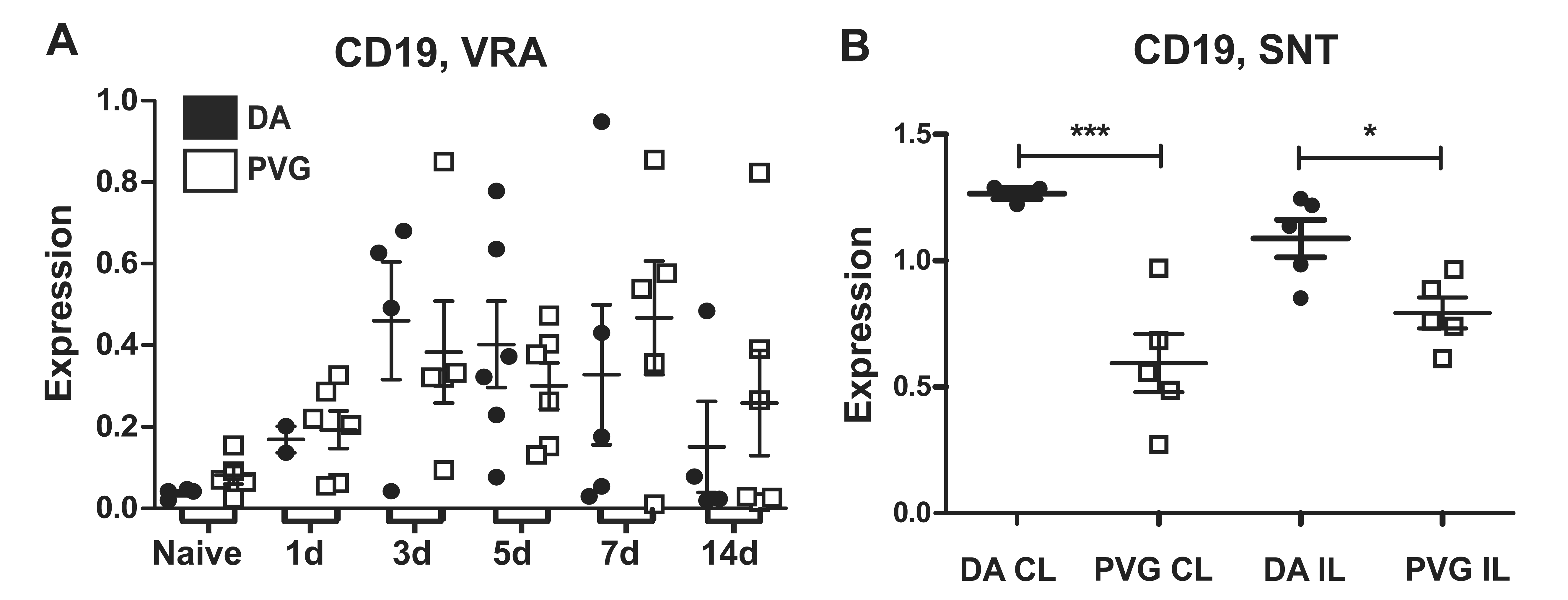

Supplement: Additional file 4: Figure S2. — Low CD19 expression following ventral root avulsion and sciatic nerve transection in rats argues against B cell infiltration. A slight up regulation of CD19 occurred after VRA injury in both DA and PVG rats, but without significant strain differences, though levels tended to be higher in DA (A) and thus opposite to the expression pattern of Cr2, n = 5–7 per strain per time-point. No difference in CD19 expression was evident between injured and control sides after SNT (B), n = 5 + 5. The results are represented as mean ± SEM. (TIFF 207 kb) [file 12974_2015_413_MOESM4_ESM.tif]
